# The Ongoing Debate Regarding Completion Thyroidectomy Versus Primary Thyroid Surgery for Low and Intermediate Differentiated Thyroid Carcinoma: A Meta-Analysis

**DOI:** 10.7759/cureus.12033

**Published:** 2020-12-11

**Authors:** Hyder Mirghani, Ibrahim A Altedlawi Albalawi

**Affiliations:** 1 Medicine, University of Tabuk, Tabuk, SAU; 2 Surgical Oncology, University of Tabuk, Tabuk, SAU

**Keywords:** lobectomy, completion thyroidectomy, differentiated thyroid cancer

## Abstract

Lobectomy is increasingly performed for low-risk differentiated thyroid cancer (DTC) and papillary thyroid microcarcinoma (PTMC). However, there is a continuous controversy about completion thyroidectomy (CT) following lobectomy. The current meta-analysis aimed to assess the outcomes of the initial surgical procedure versus CT performed for low/intermediate-risk thyroid carcinoma. Six hundred and sixty-one articles were retrieved, and only 15 full texts fulfilled the inclusion and exclusion criteria. There were 15 studies, including 17,143 patients; twelve were retrospective, two prospective studies, and a controlled trial. Seven articles were from Asia, four from the USA, two from Europe, while two were published in Canada. The studies showed no difference between lobectomy and primary thyroid surgery regarding post-surgery complications. CT was not different from the initial surgical procedure in terms of complications for DTC. The study was limited by the retrospective studies included, the outcomes assessed were not uniform, and significant heterogeneity was observed. Further, well-controlled, more specific trials are needed.

## Introduction

Differentiated thyroid cancer is the fastest increasing malignancy; completion thyroidectomy (CT) for differentiated thyroid cancer is a matter of debate and controversy over many decades [1]. The rate of CT is ranging from 5-45%, depending on the institution. The wide range of CT rates indicates the need for more discussion, research, and continuous updates. CT is not without complications; some centers reported complication rates in 14% of patients, including laryngeal nerve palsy (LNP), hypoparathyroidism (HPT), and hematomas [2]. The role of CT is mainly to facilitate radioactive iodine [3]. However, routine radioactive iodine is not recommended by most guidelines in low-risk DTC. Thyroid lobectomy is increasingly performed for differentiated low-risk thyroid carcinoma, but high rates of complications were reported, including hypothyroidism, which may be as high as 47% of patients, in addition to nerve palsy and bleeding. Previous literature concluded that radioactive iodine is needed in a considerable number of patients who underwent thyroid lobectomy, so a total thyroidectomy (TT) is needed, while others found no survival benefit in CT followed by radioactive iodine [1, 4]. Given the above controversy, an update is needed. Thus, the current meta-analysis aimed to compare the outcomes of the initial surgical procedures and CT in the management of DTC.

## Materials and methods

Eligibility criteria according to participant, intervention, control, outcome, study design (PICOS)

Type of Studies

Studies are eligible if they were conducted on humans, comparing the initial surgery (lobectomy or subtotal thyroidectomy) outcomes with CT, conducted for DTC, and published in English. No limitations for the study type and publication period were applied due to the limited previous meta-analyses. Animal studies were not included. DTC was defined as papillary or follicular without size specification.

Inclusion Criteria

All articles carried on adult humans, and in the English language from the first published article, up-to September 2019 were included in the study. The literature search was updated from 1^st^ to 31^st^ of October 2020. All retrospective, prospective cohorts, case-control studies, clinical trials, and randomized controlled studies on adults were included. The studied articles were not including any data on humans or animals. 

Type of Participants

All adults who underwent surgery for DTC with a comparison between primary surgery and CT.

Type of Intervention

Studies comparing the complications of primary thyroid surgery and CT conducted for DTC.

Outcomes Measures

The articles included in the study must compare the outcomes of CT, lobectomy, or sub-total thyroidectomy conducted for DTC, including transient or permanent hypocalcemia as measures of transient and permanent hypoparathyroidism, recurrent laryngeal nerve palsy, and recurrence and metastasis.

Exclusion Criteria

Operations on children, animal studies, free survival recurrence, and mortality were not assessed. Articles published in languages other than English, if the surgery was conducted for thyroid diseases other than DTC (nodular thyroid diseases with no concomitant malignancy, medullary and anaplastic thyroid carcinomas, autoimmune thyroid diseases, or drug-induced thyroid diseases) were excluded. The details of surgical procedures (lymph node dissection, gamma signals, robotic) were not compared. The cost and hospital stay were not included in the analysis.

Information sources, search and analysis methods

An electronic literature search was carried out in PubMed® (including Epub format and publications ahead of print), Cochrane Library, EBSCO, and Google Scholar databases. The keywords used were lobectomy, completion thyroidectomy, total thyroidectomy, differentiated thyroid cancer, primary thyroid surgery, two stages of thyroid surgery, and completion thyroidectomy followed by radioactive iodine. The protean AND and OR were used. Out of the 661 articles identified, 85 records were screened, 26 full texts were assessed, and 15 studies fulfilled the inclusion and exclusion criteria. 

**Figure 1:**
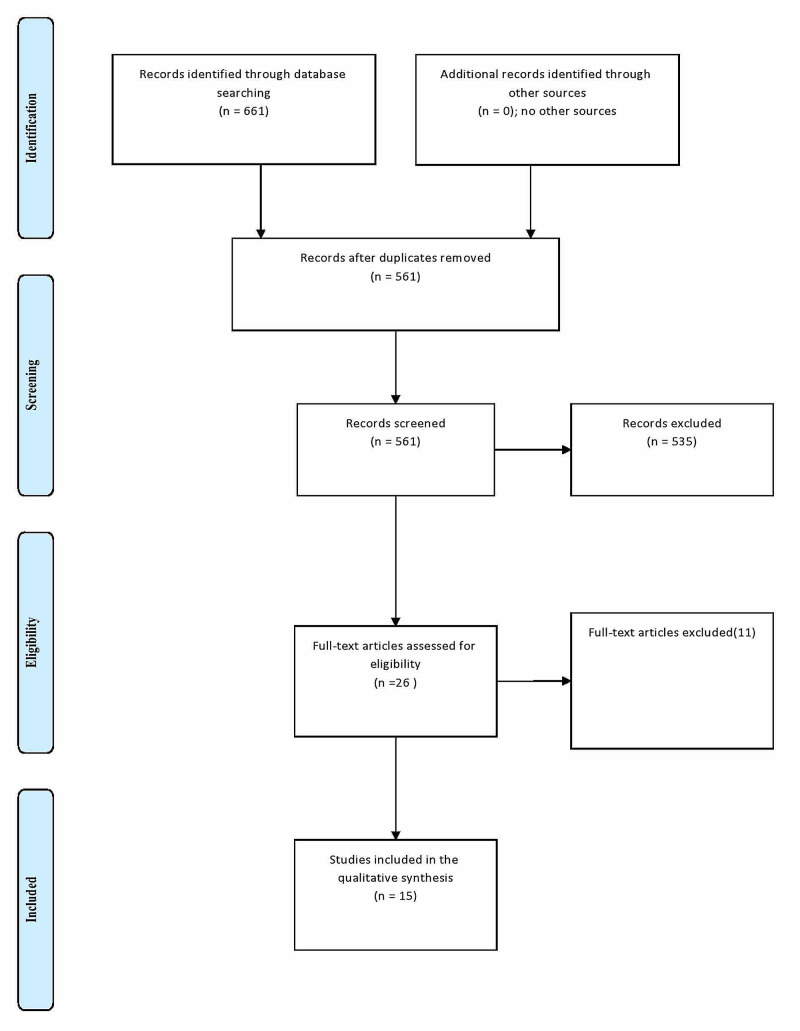
Flow diagram through the different phases of the systematic review (PRISMA flow chart) PRISMA - Preferred Reporting Items for Systematic Reviews and Meta-Analyses

Titles and abstracts were screened independently by the authors, full manuscripts relevant to the topic were then retrieved. Additional articles were searched and identified through hand searching of the bibliography. The retrieved full-text articles were assessed for eligibility for inclusion. A datasheet (including author's names, country, year of publication, study type, the number of patients included in each surgery arm, the study duration, and the reported outcomes after surgery) was used for data extraction. The datasheet was piloted on seven studies and then refined for the final use. Any disagreement in the selection of articles and data was discussed and solved between the researchers. Some of the studies were published more than once, the issue was discussed, and the results included when the outcomes were different. The retraction and republication was also a source of confusion that was resolved by discussion. The different phases of the search process are shown in Figure 1.

Statistical Analysis

The most recent RevMan (Cochrane Training, London, UK) for data analysis was used. The dichotomous data were entered manually, and the random effect was chosen due to the significant heterogeneity observed (more than 50%). The sensitivity was assessed using the funnel plot. A p-value <0.05 was considered significant.

## Results

**Figure 2:**
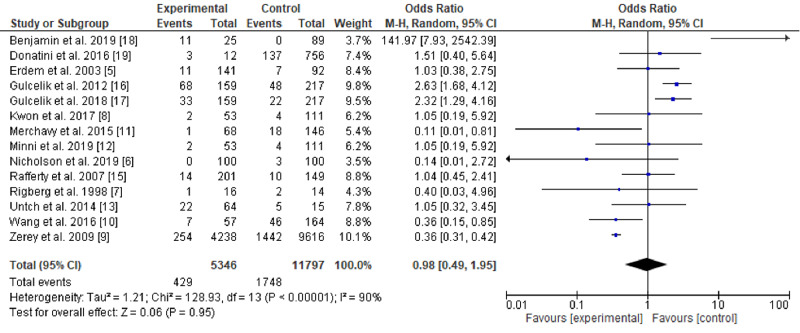
A comparison between primary thyroid surgery and completion thyroidectomy for low/intermediate-risk differentiated thyroid carcinoma M-H - Mantel-Haenszel, Tau^2 ^- cross-study variation due to heterogeneity; Chi^2 ^- Chi-square; I^2 ^- percentage of variations across studies; Z - test of the null hypothesis; df - degrees of freedom

**Figure 3:**
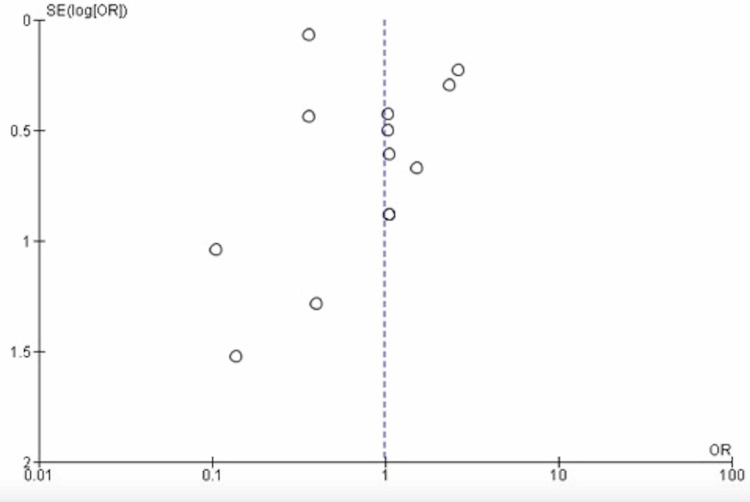
Funnel plot of total thyroidectomy versus completion thyroidectomy for low/intermediate-risk differentiated thyroid carcinoma and papillary thyroid microcarcinoma SE (log[OR]) - standard error of the log odds ratio; OR - odds ratio

Out of the 661 studies retrieved: 533 from PubMed, 100 from Google Scholar, 15 from Cochrane Library, and 2 from EBSCO); 650 studies were retrieved by including DTC keywords, while 11 studies were found when setting the searching engine for PTMC. The number stranded at 561 after the duplication removal; 26 full texts were screened, and only 15 studies fulfilled the inclusion and exclusion criteria (one study was excluded due to the zero results of complications in both arms [5], the results of another study were converted from percentages to numbers due to unavailability of the full text [6]). The studies period ranged from 3-24 years. Twelve were retrospective, two prospective studies, and one controlled trial. Seven articles were from Asia, four from the USA, two from Europe, while two were published in Canada. The present meta-analysis included 17,143 patients (11,797 in the control arm and 5,346 in the interventional group). In the current meta-analysis, five studies favored lobectomy [6-10], another five stand neutral [11-15], and four were against lobectomy [16-19]. Included studies and outcomes are presented in Tables 1-2. Significant heterogeneity was observed (90%), as showed in Figure 2. The funnel test showed significant asymmetry (Figure 3). Thus, the random effect was applied (odds ratio [OR], 0.98; 95% CI, 0.49-1.95). The overall effect was neutral, p=0.95). 

**Table 1:** Completion thyroidectomy versus primary thyroid surgery in differentiated thyroid carcinoma TT - total thyroidectomy; CT - completion thyroidectomy; RLNP - recurrent laryngeal nerve palsy; PPT - permanent hypoparathyroidism

Author	Year	Country	Type	Patients/control	Results
Erdem et al. [5]	2003	Turkey	Retrospective	11/141 lobectomy vs. 7/92 TT	No significant difference between PPT and RLNP
Nicholson et al. [6]	2019	USA	A cohort design	0/100 vs. 3/100 lobectomy vs. 7/100 for TT	Comparable outcomes
Rigberg et al. [7]	1998	USA	Prospective	1/16 vs. 2/14	CT is a safe operation
Kwon et al. [8]	2017	Korea	Retrospective	2/53vs. 4/111 TT	Lobectomy is appropriate
Zerey et al. [9]	2009	USA	Retrospective	254/4238 vs. 1442/9616	CT is associated with more morbidity
Wang et al. [10]	2016	China	Retrospective	7/57 vs. 46/164 TT	No difference in temporal hypoparathyroidism
Merchavy et al. [11]	2015	Canada	Retrospective	1/68 vs. 18/146 TT	Transient hypocalcemia is lower in CT
Minni et al. [12]	2019	Italy	Prospective	2/53 vs. 4/111 TT	The vocal alteration was higher in CT
Untch et al. [13]	2014	USA	Retrospective	22/64 vs. 5/15 lobectomy	Contralateral cancer are common
Kuşcu et al. [14]	2016	Turkey	Retrospective	0/22 vs. 0/40	CT not necessary, no recurrence
Rafferty et al. [15]	2007	Canada	Retrospective	14/201 vs. 10/149 TT	A long hospital stay for completion thyroidectomy
Gulcelik et al. 2012 [16]	2018	Turkey	Retrospective	33/159 vs. 22/217 TT	Completion thyroidectomy was shown to be as safe as a primary operation concerning permanent complications
Gulcelik et al. 2018 [17]	2012	Turkey	Trial	68/159 vs. 48/217 TT 32/271	Temporary RLNP and hypoparathyroidism are the main complications in completion thyroidectomies
Benjamin et al. [18]	2019	India	Retrospective	11/25 vs. 0/89 TT	Completion thyroidectomy is justifiable due to contralateral lobe disease and a reduction of thyroglobulin after radioactive iodine
Donatini et al. [19]	2016	France	Retrospective	3/12 vs. 0/124 lobectomy vs. 137/756 TT	Transient hypothyroidism compared

**Table 2:** The reason and outcomes of the completion thyroidectomy TH - transient hypocalcemia; PHPT - permanent hypoparathyroidism; TRLNV - transient recurrent laryngeal palsy; PRLNV - permanent recurrent laryngeal palsy; LGDTC - low-grade differentiated thyroid carcinoma; DTC - differentiated thyroid carcinoma; LGDTC - low-grade differentiated thyroid carcinoma; PTMC - papillary thyroid microcarcinoma

Author	Study period	Reason for initial surgery	Outcomes measured
Untch et al. [5]	10 years records	LGDTC	Oncologic outcomes
Merchavy et al. [6]	5 years of records	DTC	Transient hypocalcemia and permanent hypoparathyroidism
Rigberg et al. [7]	Not stated	DTC	Transient hypocalcemia, and transient recurrent laryngeal palsy
Erdem et al. [8]	8 years	DTC	Permanent hypoparathyroidism and recurrent laryngeal palsy
Rafferty et al. [9]	10 years records	Multinodular goiter	Permanent hypoparathyroidism and recurrent laryngeal palsy
Zerev et al. [10]	5 years	DTC	Cost and hospital stay
Gulcelik et al. 2012 [11]	5 years of records	DTC	Transient hypocalcemia, permanent hypoparathyroidism, and recurrent laryngeal palsy
Donatini et al. [12]	24 years records	PTMC	Transient hypocalcemia and transient recurrent laryngeal palsy
Wang et al. [13]	3 years of records	DTC	Transient hypocalcemia
Gulcelik et al. 2012 [14]	10 years	DTC	Transient hypocalcemia, permanent hypoparathyroidism, and recurrent laryngeal palsy
Kuşcu et al. [15]	15 years records	PTMC	Recurrence
Kwon et al. [16]	8.5 years	PTMC	Transient hypocalcemia, permanent hypoparathyroidism, and recurrence-free survival
Nicholson et al. [17]	10 years	DTC	Transient hypocalcemia, permanent hypoparathyroidism, and recurrent laryngeal palsy
Benjamin et al. [18]	6 years of records	LGDTC	Recurrence rate
Minni et al. [19]	2 years prospective	Multinodular or LGDTC	External branch of superior laryngeal nerve injury

## Discussion

Differentiated thyroid carcinoma is rising worldwide, mainly due to the increase in screening and overdiagnosis. The increasing rate of DTC is mirrored by increasing both primary and completion thyroidectomy [20, 21]. The present data showed that the initial operation (total thyroidectomy [TT] or lobectomy) carries the same outcomes in transient hypocalcemia (TH), permanent hypoparathyroidism (PHPT), recurrent laryngeal nerve (RCLN), and RCLN injury. However, the evidence is weak because most of the included studies were retrospective. Besides, the significant heterogeneity observed might affect the result. Our findings supported a previous meta-analysis published by Li and colleagues, which assessed transient and permanent hypocalcemia, RLN injury, hematoma, and wound infection. Li et al. study is limited by the small number of the studies included, the limited databases searched, and the period of their search [18]. Our result implies that the surgeon might choose either lobectomy with careful follow-up for recurrence or total thyroidectomy. A careful patient's assessment for risk stratification, a well-informed, preference-based decision, and the surgeon experience might direct the extent of the initial thyroid surgery for DTC [22, 23].

Reviews and meta-analyses evaluating completion thyroidectomy versus the initial surgery are scarce. Thyroid surgeries are increasingly performed due to improving diagnostic techniques, screening, or true increasing rates [24, 25]. In addition, the high body mass index has dramatically increased and was found to be associated with DTC in a large case-control study (frequency-matched) that included 10,668 DTC patients versus 11,858 control [26]. Given the above, an update is highly needed.

There are several limitations of the current study. We included both prospective and retrospective studies; the included studies cannot control for various confounders that may affect the outcomes, including the original thyroid disease, the patient's age, comorbidities, and the extent of surgery, including central lymph nodes dissection [27]. Besides, a high-volume surgical team might affect the outcomes [28]. Also, the limitations include the significant observed heterogeneity and studies published only in the English language.

## Conclusions

Completion thyroidectomy was comparable to initial thyroid surgery (in terms of transient or permanent hyperparathyroidism and recurrent laryngeal nerve injury) when applied for differentiated thyroid cancer and papillary thyroid microcarcinoma. The surgeon might choose between total thyroidectomy and lobectomy as initial surgery for the same disease. The current data gave an insight into the current knowledge regarding thyroid surgery for low-grade differentiated thyroid carcinoma and papillary thyroid microcarcinoma. It was limited by the retrospective studies included, the outcomes assessed were not uniform, and significant heterogeneity was observed. Further, well-controlled, more specific trials are needed.
